# Extracorporeal Cytokine Hemadsorption with oXiris^®^ in Critically Ill Patients with Non-Septic Vasoplegic Shock: Hemodynamic Effects, Cytokine Kinetics, and Mortality Outcomes

**DOI:** 10.3390/jcm15124414

**Published:** 2026-06-07

**Authors:** Hakan Küçükkepeci, Sinan Mutlu, Rasim Onur Karaoğlu, Açelya Toprak Karaoğlu, Özge Sayın, Namigar Turgut

**Affiliations:** 1 Department of Anesthesiology and Reanimation, Şırnak Cizre Dr. Selahattin Cizrelioğlu State Hospital, Şırnak 73200, Türkiye; hakankucukkepeci@gmail.com (H.K.); ssinanmutlu@gmail.com (S.M.); 2 Department of Anesthesiology and Reanimation, SBÜ Bağcılar Training and Research Hospital, Istanbul 34200, Türkiye; 3 Department of Anesthesiology and Reanimation, Zeynep Kamil Maternity and Children’s Hospital, Istanbul 34668, Türkiye; 4 Department of Intensive Care, Ege University Faculty of Medicine, Izmir 35100, Türkiye; 5 Department of Anesthesiology and Reanimation, Prof. Dr. Cemil Taşcıoğlu City Hospital, Istanbul 34384, Türkiye

**Keywords:** vasoplegic shock, cytokine hemadsorption, oXiris^®^, extracorporeal blood purification, cytokine storm, intensive care unit, sepsis, mortality

## Abstract

**Background**: Vasoplegic shock (VS) in critically ill patients without microbiological evidence of infection poses a major clinical challenge in intensive care units (ICUs). Extracorporeal cytokine hemadsorption using the oXiris^®^ membrane—a high-permeability polyacrylonitrile-based (AN69-ST) filter with adsorptive properties against inflammatory mediators—has emerged as a potential adjunct to restore haemodynamic stability. Evidence supporting its use remains limited, particularly regarding timing of initiation and downstream mortality biomarkers. **Methods**: We conducted a single-centre prospective observational study at the ICU of Istanbul Prof. Dr. Cemil Taşcıoğlu City Hospital between October 2022 and January 2023. Adults aged ≥18 years with VS (CRP ≥ 100 mg/L, procalcitonin [PCT] < 2 μg/L, no positive microbiological culture) requiring continuous renal replacement therapy (CRRT) with the oXiris^®^ membrane were enrolled (*n* = 34), of whom 30 completed the study period without microbiological exclusion and comprised the final analysis cohort. Pre- and post-treatment (72 h) clinical and cytokine parameters were compared. The association of VS resolution and 7-day mortality with timing of oXiris^®^ initiation, cytokine levels, and treatment duration was assessed. This study was conducted and reported in accordance with the STROBE (Strengthening the Reporting of Observational Studies in Epidemiology) statement. **Results**: Significant changes were observed across all principal haemodynamic and inflammatory parameters at 72 h of oXiris^®^ treatment, including mean arterial pressure (MAP: 50.8 ± 6.3 to 69.3 ± 17 mmHg, *p* < 0.001), SOFA score (8.33 ± 2.29 to 4.9 ± 3.22, *p* < 0.001), IL-6 (767.3 ± 1205.7 to 294.4 ± 686.3 pg/mL, *p* < 0.001), IL-1β, TNF-α, CRP, lactate, and creatinine. VS resolved in 24/30 patients (80%). Younger age was associated with VS resolution (57.8 ± 19.7 vs. 76.6 ± 13.9 years; *p* = 0.021). Initiation of oXiris^®^ within 8 h was associated with significantly shorter VS resolution time (52.5 ± 23.9 vs. 85.9 ± 48.2 h; *p* = 0.045). Seven-day mortality was 20% (n = 6) and hospital mortality was 50% (n = 15). Post-treatment IL-1β (856.7 ± 548.5 vs. 1086.9 ± 353.6 pg/mL; *p* = 0.044) and TNF-α (111.0 ± 70.0 vs. 145.4 ± 47.8 pg/mL; *p* = 0.011) at 72 h were significantly higher in hospital non-survivors, representing exploratory prognostic associations. **Conclusions**: Changes in haemodynamic and inflammatory parameters were observed during oXiris^®^-based CRRT treatment in critically ill patients with non-septic VS. Early initiation (≤8 h) was associated with shorter VS resolution time in this exploratory, uncontrolled analysis. Residual IL-1β and TNF-α at 72 h were associated with hospital mortality in exploratory analyses and may represent hypothesis-generating prognostic signals requiring prospective validation. Randomised controlled trials are warranted to confirm these findings and define optimal timing strategies.

## 1. Introduction

Vasoplegic shock (VS) is characterised by pathologically low systemic vascular resistance (SVR) despite normal or elevated cardiac output, resulting in refractory hypotension and end-organ hypoperfusion [1,2]. Although sepsis represents the most frequent precipitant in the ICU setting, VS can arise in the absence of confirmed infection across a spectrum of clinical scenarios including post-cardiopulmonary bypass surgery, major trauma, burns, and severe systemic inflammatory response syndrome [1,3]. This non-infectious, cytokine-driven variant of VS poses unique diagnostic and therapeutic challenges; standard vasopressor escalation frequently fails to restore haemodynamic stability, and evidence-based adjuncts remain sparse. The reported incidence of vasoplegic shock varies considerably by clinical context, ranging from 5–44% in post-cardiac surgery patients and carrying associated mortality rates of 25–50% [4,5]. Beyond the surgical setting, vasopressor-dependent distributive shock in the broader ICU population is associated with similarly high mortality, with Turkish multicentre data reporting ICU mortality rates of approximately 46% in patients with severe shock and organ failure [6]. Population-specific data on non-infectious VS in Turkish ICUs remain scarce, underscoring the need for locally applicable evidence to guide its management.

The pathophysiology of VS centres on dysregulated release of pro-inflammatory cytokines—most prominently interleukin-6 (IL-6), IL-1β, tumour necrosis factor-alpha (TNF-α), and IL-10—which collectively promote nitric oxide (NO)-mediated vasodilatation, impair vascular smooth muscle responsiveness, and trigger downstream organ dysfunction [3,7]. High circulating cytokine concentrations are associated with increased severity of organ failure and mortality [8,9]. Early identification and attenuation of this hyperinflammatory state is therefore paramount.

Extracorporeal cytokine hemadsorption has emerged as a promising immunomodulatory strategy in the critically ill. The oXiris^®^ membrane (Baxter International), a polyacrylonitrile (AN69) filter coated with positively charged polyethyleneimine, adsorbs circulating cytokines, endotoxins, and other pro-inflammatory mediators through electrostatic and hydrophobic interactions during CRRT [10,11]. Preclinical and early clinical data suggest that oXiris^®^ can reduce cytokine burden, attenuate vasopressor requirements, and potentially improve organ function in septic and post-cardiac surgery patients [12,13,14,15]. However, robust evidence specifically addressing non-infectious VS, the optimal timing of initiation, and the prognostic value of residual cytokine levels post-treatment is lacking.

We therefore designed a prospective observational study to describe haemodynamic and cytokine-kinetic changes observed during oXiris^®^-based CRRT and to explore associations between treatment timing, cytokine trajectories, and clinical outcomes in critically ill patients with non-microbiological VS, with particular emphasis on the relationship between timing of initiation and clinical response, and the prognostic value of post-treatment cytokine levels for mortality.

## 2. Methods

### 2.1. Study Design and Setting

This was a single-centre, prospective observational cohort study conducted in the ICU of Istanbul Prof. Dr. Cemil Taşcıoğlu City Hospital, University of Health Sciences, between 1 October 2022 and 1 January 2023. The study was approved by the institutional ethics committee and conducted in accordance with the Declaration of Helsinki. Written informed consent was obtained from patients or their legally authorised representatives. Reporting followed the STROBE checklist for observational studies [16].

### 2.2. Participants

Consecutive adult patients (aged ≥ 18 years) admitted to the ICU with VS were screened for eligibility. VS was defined as persistent hypotension (MAP < 65 mmHg) requiring vasopressors despite adequate fluid resuscitation, in the context of a systemic inflammatory response characterised by CRP ≥ 100 mg/L and PCT < 2 μg/L, and with no positive microbiological culture (blood, urine, or respiratory secretions) at the time of enrolment [1,17]. Beyond microbiological cultures, sepsis was further excluded through clinical assessment by the attending intensivist, including physical examination for an identifiable infectious focus (including fever pattern, localising signs of infection, wound assessment, and indwelling catheter sites) and review of available imaging. Patients in whom occult infection could not be confidently excluded on clinical and biochemical grounds were not enrolled. All eligible patients meeting inclusion criteria were commenced on CRRT using the oXiris^®^ membrane (Baxter Healthcare Corporation, Deerfield, IL, USA). The decision to initiate oXiris^®^ (Baxter Healthcare Corporation, Deerfield, IL, USA) was made by the attending intensivist based on the presence of refractory vasoplegic shock despite standard vasopressor therapy, in the absence of microbiological evidence of infection. Patients with active haematological malignancy, pregnancy, life-limiting illness (anticipated survival < 24 h), or previous participation in the study were excluded. Of 34 patients initially screened and enrolled, 4 were subsequently excluded following positive microbiological cultures obtained during routine weekly surveillance or clinically indicated repeat sampling, leaving 30 patients for final analysis. No patients among the final 30 developed positive cultures during the study period. No antibiotics were administered prior to culture collection. Viral and fungal infections were excluded through standard diagnostic workup including viral PCR panels, galactomannan, and beta-glucan assays where clinically indicated. The underlying aetiologies of non-infectious VS in the final cohort were: post-cardiac surgery (n = 7, 23.3%), burns (n = 5, 16.7%), major trauma (n = 5, 16.7%), adrenal insufficiency (n = 4, 13.3%), pancreatitis (n = 3, 10.0%), drug/toxin exposure (n = 3, 10.0%), and anaphylaxis (n = 3, 10.0%).

### 2.3. oXiris^®^ Treatment Protocol

All patients received standardised ICU care comprising fluid resuscitation, norepinephrine (NE) titrated to a MAP target ≥ 65 mmHg, mechanical ventilation with a lung-protective strategy where applicable, nutritional support, and haemodynamic monitoring. Corticosteroid use (hydrocortisone 200 mg/day) was administered at the attending intensivist’s discretion in cases of refractory vasoplegic shock, and was documented for each patient. No other vasoactive agents or immunomodulatory therapies were administered during the study period that would be expected to independently influence cytokine kinetics or haemodynamic recovery. oXiris^®^-based CRRT was initiated using continuous veno-venous haemodiafiltration (CVVHDF) mode. Blood flow rate was maintained at 150–200 mL/min, with a mean ultrafiltration rate of 66.6 ± 28.8 mL/h. Treatment duration was determined at the clinician’s discretion based on haemodynamic response and cytokine trajectory. For analytical purposes, patients were stratified according to whether oXiris^®^ was initiated within 8 h of ICU admission or thereafter. This threshold was not prespecified in the study protocol but was selected post hoc based on the observed distribution of initiation times in the cohort.

### 2.4. Data Collection and Outcomes

Baseline and 72-h post-initiation measurements included: body temperature, MAP, heart rate, respiratory rate, Glasgow Coma Scale (GCS), NE dose, arterial lactate, creatinine, alanine aminotransferase (ALT), procalcitonin (PCT), C-reactive protein (CRP), haemogram (haemoglobin, platelets, white blood cell count, neutrophil percentage), PaO_2_/FiO_2_ ratio, and cytokines (IL-6, IL-1β, TNF-α, IL-10) measured by enzyme-linked immunosorbent assay (ELISA). Organ dysfunction was quantified using the Sequential Organ Failure Assessment (SOFA) score [18].

The primary outcomes were: (i) VS resolution (defined as liberation from vasopressors and maintenance of MAP ≥ 65 mmHg without vasoactive support for ≥24 consecutive hours), (ii) 7-day ICU mortality, and (iii) hospital mortality at discharge. Secondary outcomes included changes in haemodynamic and inflammatory parameters at 72 h, time to VS resolution, and factors associated with non-resolution and mortality.

### 2.5. Statistical Analysis

Continuous variables are reported as mean ± standard deviation (SD). Categorical variables are expressed as frequencies and percentages. Comparison of pre- and post-treatment parameters was performed using the Wilcoxon signed-rank test for non-normally distributed data and the paired t-test where appropriate. Between-group comparisons were made using the Mann-Whitney U test for continuous variables and Fisher’s exact test for categorical variables. A two-tailed *p* value < 0.05 was considered statistically significant. Statistical analyses were performed using IBM SPSS Statistics version 26.0 (IBM Corp., Armonk, NY, USA). Given the exploratory nature of this study and the multiple comparisons performed across clinical, laboratory, and cytokine variables, no adjustment for multiple testing was applied. All analyses should be interpreted as hypothesis-generating, and findings with *p* values close to the 0.05 threshold should be interpreted with particular caution.

## 3. Results

### 3.1. Patient Characteristics

Of 34 patients initially enrolled, 4 were subsequently excluded following positive microbiological cultures, leaving 30 patients for final analysis. The mean age was 61.6 ± 19.9 years (range 21–91); 16 (53.3%) were male and 14 (46.7%) female. Mean ICU length of stay was 27.5 ± 26.4 days. oXiris^®^ membrane therapy was initiated at a mean of 10.3 ± 7.8 h following ICU admission. Baseline cytokine levels were markedly elevated: IL-6 767.3 ± 1205.7 pg/mL, IL-1β 1553.0 ± 800.7 pg/mL, TNF-α 211.9 ± 95.5 pg/mL, and IL-10 308.2 ± 141.0 pg/mL, consistent with a severe hyperinflammatory phenotype. Baseline patient characteristics and underlying aetiologies are summarised in Table 1.

### 3.2. Haemodynamic and Inflammatory Outcomes at 72 h

Statistically significant changes were observed across all principal haemodynamic and inflammatory indices at 72 h of oXiris^®^ treatment (Table 2). MAP increased from 50.8 ± 6.3 to 69.3 ± 17.0 mmHg (*p* < 0.001) and NE dose decreased from 0.29 ± 0.35 to 0.17 ± 0.32 μg/kg/min (*p* = 0.011). Heart rate declined from 117.7 ± 19.5 to 88 ± 20 bpm (*p* < 0.001) and respiratory rate from 20 ± 4.8 to 15.6 ± 3 breaths/min (*p* < 0.001). The PaO_2_/FiO_2_ ratio improved from 267.8 ± 97 to 354.3 ± 111.8 (*p* < 0.001) and hourly urine output from 62.8 ± 51.6 to 94.1 ± 64.5 mL/h (*p* < 0.001). SOFA score decreased from 8.33 ± 2.29 to 4.90 ± 3.22 (*p* < 0.001), and creatinine from 2.33 ± 1.63 to 0.97 ± 0.74 mg/dL (*p* < 0.001), reflecting broad multi-organ recovery. All measured cytokines, CRP, PCT, and lactate declined significantly (all *p* < 0.05); ALT did not reach statistical significance (*p* = 0.11).

### 3.3. Vasoplegic Shock Resolution and the Role of Age

VS resolved in 24 of 30 patients (80.0%). Patients whose VS resolved were significantly younger than those who did not resolve (57.8 ± 19.7 vs. 76.6 ± 13.9 years; *p* = 0.021) (Table 3). This association should be interpreted as unadjusted; whether age per se or associated differences in physiological reserve and comorbidity burden underlie this relationship cannot be determined without multivariable analysis. Time to oXiris^®^ initiation did not significantly differ between resolvers and non-resolvers (9.42 ± 4.6 vs. 12.17 ± 12.44 h; *p* = 0.616).

### 3.4. Impact of Timing of oXiris^®^ Initiation

Stratification by timing of oXiris^®^ initiation revealed differences in VS resolution time between groups in this exploratory analysis. Patients in whom treatment was commenced within 8 h of ICU admission (n = 16) achieved VS resolution significantly faster than those in whom treatment was delayed beyond 8 h (n = 14): 52.5 ± 23.9 vs. 85.9 ± 48.2 h (*p* = 0.045). IL-6 levels at 72 h did not significantly differ between early and delayed initiation groups (373.9 ± 827.3 vs. 203.6 ± 494.5 pg/mL; *p* = 0.114). NE dose reduction at 72 h was numerically greater in the early-initiation group but did not reach statistical significance (*p* = 0.594). Baseline characteristics were well-matched between groups, with the exception of IL-6, which was numerically higher in the early-initiation group (1053.4 ± 1419.5 vs. 440.6 ± 838.2 pg/mL; *p* = 0.048). Full baseline and outcome comparisons are presented in Table 4.

### 3.5. Mortality and Predictive Cytokine Biomarkers

Seven-day ICU mortality occurred in 6 patients (20%). Hospital mortality was 50% (n = 15). Seven-day non-survivors were older (76.7 ± 13.9 vs. 57.9 ± 19.7 years; *p* = 0.040), had higher baseline NE dose (0.6 ± 0.6 vs. 0.2 ± 0.2 μg/kg/min; *p* = 0.014) and lactate (6.6 ± 2.4 vs. 3.8 ± 1.9 mmol/L; *p* = 0.016). Notably, all 6 seven-day non-survivors were among patients whose VS did not resolve, indicating complete overlap between these two groups. Baseline cytokine levels did not significantly differ between survivors and non-survivors. Patients who died within 7 days had significantly lower total oXiris^®^ treatment duration compared with survivors (Table 5). This association should be interpreted with caution: shorter treatment duration in non-survivors likely reflects earlier death rather than a causal relationship between treatment exposure and mortality—a form of survivorship bias inherent to observational studies of this design. Whether prolonged oXiris^®^ therapy would have altered outcomes cannot be determined from this data. Baseline cytokine levels did not predict 7-day mortality, with no significant differences between survivors and non-survivors for IL-6, IL-1β, TNF-α, or IL-10 (all *p* > 0.2, Table 5), supporting the hypothesis that post-treatment cytokine trajectories rather than pre-treatment levels carry prognostic information. Critically, IL-6 levels at 72 h post-initiation were markedly higher in 7-day non-survivors: 1052.5 ± 1352.9 vs. 104.9 ± 92.9 pg/mL (*p* = 0.026).

With respect to hospital mortality, IL-1β and TNF-α concentrations at 72 h were both significantly higher in patients who ultimately died before discharge (Table 6). IL-1β was 1086.9 ± 353.6 pg/mL in non-survivors versus 856.7 ± 548.5 pg/mL in survivors (*p* = 0.044), and TNF-α was 145.4 ± 47.8 vs. 111.0 ± 70.0 pg/mL (*p* = 0.011). These findings suggest that residual IL-1β and TNF-α at 72 h may represent exploratory prognostic signals for hospital-level mortality, though formal validation in larger cohorts is required before these can be considered established biomarkers.

## 4. Discussion

To our knowledge, this is among the first prospective observational studies specifically targeting non-infectious VS—a clinically distinct entity characterised by cytokine-driven haemodynamic collapse without microbiological confirmation—to characterise the haemodynamic and cytokine-kinetic effects of oXiris^®^-based CRRT, and to link treatment timing and residual cytokine trajectories to clinical outcomes.

The principal finding of this study is that broad, statistically significant changes across haemodynamic and inflammatory parameters were observed at 72 h during oXiris^®^ hemadsorption treatment, including MAP, heart rate, respiratory rate, SOFA score, creatinine, oxygenation, and all four measured cytokines. These findings are concordant with the mechanistic rationale for AN69-ST membranes: the positively charged polyethyleneimine coating facilitates high-affinity electrostatic adsorption of cytokines, endotoxin, and other charged inflammatory mediators [10,19]. The magnitude of cytokine reductions observed here—IL-6 declining by 62%, TNF-α by 40%, IL-1β by 37%, and CRP by 46%—is consistent with findings from prior studies in septic populations, including the randomised crossover trial by Broman et al. [14] and the multicentre experience reported by Schwindenhammer et al. [13].

A fundamental observation of prognostic and pathophysiological relevance is the association between younger age and VS resolution. Patients whose VS resolved were substantially younger (57.8 vs. 76.6 years; *p* = 0.021). Age-related immune senescence and diminished vascular plasticity likely underlie this gradient; elderly patients may exhibit a blunted compensatory vasomotor response even after cytokine burden is reduced [20]. Specifically within the vasoplegic shock literature, Roscani et al. identified older age as an independent predictor of severe vasodilatory shock following cardiac surgery, with each additional year of age conferring a measurable increase in risk (OR = 1.04; *p* = 0.016), underscoring the particular vulnerability of elderly patients to vasomotor dysregulation under conditions of systemic inflammatory stress [21]. This finding has direct clinical implications: older patients may require more prolonged or intensified extracorporeal support, or combination strategies addressing both cytokine excess and vascular hypo-responsiveness. These observations are consistent with a broader body of literature linking advancing age to poorer outcomes in vasopressor-dependent shock. Marin-Corral et al. demonstrated that older age was independently associated with increased ICU mortality across a large multicentre sepsis cohort, with each decade of age conferring a measurable incremental increase in risk [22]. Similarly, Guidet et al. reported that physiological reserve and frailty—both of which decline progressively with age—were stronger determinants of shock non-resolution than acute illness severity scores alone [23]. In the context of extracorporeal immunomodulation specifically, aged immune cells exhibit dysregulated cytokine production kinetics and a diminished capacity for homeostatic recovery following cytokine depletion, which may attenuate the haemodynamic benefit of oXiris^®^ therapy in older patients [24]. Prospective evaluation of age-stratified treatment protocols or extended filter duration in elderly patients is therefore warranted.

An exploratory association between earlier oXiris^®^ initiation and shorter VS resolution time was observed. Patients in whom therapy was commenced within 8 h of ICU admission achieved VS resolution in approximately half the time compared to those treated after 8 h (52.5 vs. 85.9 h; *p* = 0.045). 72-h IL-6 levels did not significantly differ between timing groups (*p* = 0.114), though VS resolution time remained significantly shorter in the early-initiation group. These data are consistent with the hypothesis that extracorporeal cytokine removal may be most efficacious when deployed before the cytokine cascade reaches a self-amplifying threshold—analogous to the established principle in septic shock that early goal-directed resuscitation improves outcomes [25,26]. Our findings parallel those of studies in cardiopulmonary bypass patients demonstrating that early post-operative cytokine adsorption reduces vasopressor dependency and ICU length of stay [27]. It should be noted that the 8-h threshold used in this analysis was not prespecified and has no established precedent in the literature, where early’ initiation is variably defined as within 6–24 h of shock onset. This subgroup analysis should therefore be interpreted as exploratory and hypothesis-generating, pending validation in prospective trials with a prespecified timing threshold.

The prognostic biomarker findings deserve particular attention. The observation that 72-h IL-6 levels were higher in 7-day non-survivors compared with survivors and that 72-h IL-1β and TNF-α differentiated hospital survivors from non-survivors, suggests that post-treatment cytokine trajectories—not merely pre-treatment levels—carry exploratory prognostic information. We hypothesise, in a purely exploratory framework, that oXiris^®^ may serve a dual role as both a therapeutic intervention and an indirect immunological probe: the extent of sustained cytokine reduction may reflect the patients capacity for immune homeostasis. This concept remains speculative and requires prospective validation before clinical application. This aligns with emerging data from sepsis biomarker research demonstrating that kinetic changes in IL-6 and IL-1β over the first 72 h of ICU admission are superior prognostic markers to single baseline measurements [9,28]. Residual TNF-α elevation may further reflect macrophage hyperactivation and endothelial damage that persist beyond the acute cytokine surge [29].

Our 7-day mortality rate of 20% and hospital mortality of 50% should be interpreted cautiously in the absence of a comparator group. However, these figures are broadly consistent with reported mortality rates in comparable ICU populations with severe haemodynamic instability requiring vasopressors, which range from 30–60% depending on case mix and illness severity [30]. The relatively lower 7-day mortality is consistent with the haemodynamic changes observed during oXiris^®^ therapy, though causal inference cannot be drawn from this uncontrolled observation, while the higher hospital mortality underscores that immune dysregulation-associated organ injury continues to accrue beyond 7 days, potentially beyond the window of extracorporeal cytokine removal.

### Strengths and Limitations

The strengths of this study include its prospective design, consistent treatment protocol, comprehensive cytokine profiling at defined time points, and clinically meaningful outcome stratification by timing and survival. The study period was defined by institutional oXiris^®^ availability and resource constraints. Given the stringent inclusion criteria—particularly the requirement for confirmed non-infectious VS based on negative microbiological cultures and PCT below 2 μg/L—the recruitment of 34 initially eligible patients, of whom 30 comprised the final cohort following microbiological exclusion reflects the incidence of this clinical phenotype in our ICU. This study was therefore designed as a hypothesis-generating pilot investigation to characterise cytokine kinetics and haemodynamic responses, with findings intended to inform the design of future adequately powered randomised controlled trials. Limitations include the single-centre design, small sample size (n = 30), and absence of a contemporaneous control group—the most fundamental constraint of this study. In the absence of a control arm, all observed changes in haemodynamic and cytokine parameters must be interpreted as descriptive observations rather than treatment effects, since spontaneous disease trajectory cannot be excluded. Furthermore, multiple comparisons were performed across clinical, laboratory, and cytokine variables without correction for multiplicity; accordingly, all *p* values should be interpreted as hypothesis-generating rather than confirmatory, and findings with *p* values close to the 0.05 threshold warrant particular caution. The heterogeneity of underlying conditions leading to non-infectious VS may limit generalisability. Adjustment for confounding factors was limited throughout; all reported associations between age, timing of initiation, cytokine levels, and clinical outcomes are unadjusted and may reflect underlying differences in illness severity, comorbidity burden, or aetiology rather than independent effects of the variables examined. Multivariable analyses were not feasible given the sample size. Longer-term follow-up data and quality of life assessments were not collected. Treatment duration varied considerably across patients and was determined at the clinician’s discretion rather than by a predefined protocol, which may confound comparisons between groups. Potential selection bias arises from the pragmatic, clinician-directed allocation of oXiris^®^ initiation timing, which was not governed by a prespecified protocol and may reflect unmeasured differences in illness trajectory or clinical judgement. Survivorship bias is a further explicit concern: patients who died early had shorter total treatment exposure, which may artificially inflate apparent associations between treatment duration and survival outcomes, and precludes causal interpretation of this relationship. Furthermore, in the absence of a control group, it cannot be excluded that the observed reductions in cytokine levels and haemodynamic improvements partially reflect the natural trajectory of systemic inflammation in critically ill patients, independent of extracorporeal adsorption. Spontaneous cytokine clearance and vasomotor recovery may occur over a similar timeframe in some patients with non-infectious VS, and the relative contribution of oXiris^®^ therapy to the observed improvements cannot be definitively quantified without a randomised comparator arm. These limitations notwithstanding, the study provides important hypothesis-generating data to inform the design of adequately powered randomised controlled trials.

## 5. Conclusions

In critically ill patients with non-infectious vasoplegic shock, changes consistent with haemodynamic stabilisation, reduced cytokine burden, and attenuated multi-organ dysfunction were observed during oXiris^®^-based CRRT over 72 h. Older age was associated with failure to resolve VS. Early initiation of hemadsorption (within 8 h of ICU admission) was associated with shorter time to VS resolution. Residual IL-1β and TNF-α concentrations at 72 h post-treatment were associated with hospital mortality in exploratory analyses. These hypothesis-generating findings are consistent with the principle of early extracorporeal cytokine removal, though causal inference is precluded by the absence of a control group in hyperinflammatory non-septic VS and warrant prospective randomised validation in larger, multicentre cohorts.

## Figures and Tables

**Table 1:** Baseline Patient Characteristics (n = 30).

Variable	Total Cohort (n = 30)
Age, years (mean ± SD)	61.6 ± 19.9
Age range, years	21–91
Male, n (%)	16 (53.3%)
Female, n (%)	14 (46.7%)
ICU length of stay, days (mean ± SD)	27.5 ± 26.4
Underlying aetiology, n (%)	
Post-cardiac surgery	7 (23.3%)
Burns	5 (16.7%)
Major trauma	5 (16.7%)
Adrenal insufficiency	4 (13.3%)
Pancreatitis	3 (10.0%)
Drug/toxin exposure	3 (10.0%)
Anaphylaxis	3 (10.0%)

Data are mean ± SD or n (%). ICU, intensive care unit.

**Table 2:** Changes in Clinical and Laboratory Parameters Before and 72 h After oXiris^®^ Initiation (n = 30).

Variable	Pre-oXiris^®^	Post-oXiris^®^	*p* Value
Body temperature (°C)	37.4 ± 0.8	36.2 ± 0.2	<0.001
MAP (mmHg)	50.8 ± 6.3	69.3 ± 17	<0.001
Heart rate (bpm)	117.7 ± 19.5	88 ± 20	<0.001
Respiratory rate (breaths/min)	20 ± 4.8	15.6 ± 3	<0.001
NE dosage (μg/kg/min)	0.29 ± 0.35	0.17 ± 0.32	0.011
PCT (μg/L)	1.03 ± 0.6	0.58 ± 0.33	<0.001
Lactate (mmol/L)	4.36 ± 2.31	2.53 ± 2.75	0.001
Urine output (mL/h)	62.8 ± 51.6	94.1 ± 64.5	<0.001
SOFA score	8.33 ± 2.29	4.9 ± 3.22	<0.001
Haemoglobin (g/dL)	10.4 ± 2.35	9.2 ± 1.8	<0.001
Platelets (×10^3^/mm^3^)	196.8 ± 98.9	137.9 ± 85.8	<0.001
WBC (×10^3^/mm^3^)	14.6 ± 6.4	12.7 ± 7.3	0.021
Neutrophils (%)	86.3 ± 5	78.9 ± 10.7	<0.001
Creatinine (mg/dL)	2.33 ± 1.63	0.97 ± 0.74	<0.001
ALT (U/L)	86.8 ± 128.5	61 ± 115	0.11
PaO_2_/FiO_2_ ratio	267.8 ± 97	354.3 ± 111.8	<0.001
IL-6 (pg/mL)	767.3 ± 1205.7	294.4 ± 686.3	<0.001
IL-1β (pg/mL)	1553.0 ± 800.7	971.8 ± 468.3	<0.001
TNF-α (pg/mL)	211.9 ± 95.5	128.2 ± 61.4	<0.001
IL-10 (pg/mL)	308.2 ± 141.0	225.8 ± 86.0	<0.001
CRP (mg/L)	226.6 ± 103.3	122.6 ± 71.7	<0.001

Data are mean ± SD. ALT, alanine aminotransferase; CRP, C-reactive protein; FiO_2_, fraction of inspired oxygen; IL, interleukin; MAP, mean arterial pressure; NE, norepinephrine; PaO_2_, partial pressure of arterial oxygen; PCT, procalcitonin; SOFA, Sequential Organ Failure Assessment; TNF-α, tumour necrosis factor-alpha; VS, vasoplegic shock; WBC, white blood cell count.

**Table 3:** Age and Time to oXiris^®^ Initiation According to Vasoplegic Shock Resolution.

Variable	VS Resolved (n = 24)	VS Not Resolved (n = 6)	*p* Value
Age (years)	57.8 ± 19.7	76.6 ± 13.9	0.021
Time to oXiris^®^ initiation (h)	9.42 ± 4.6	12.17 ± 12.44	0.616

Data are mean ± SD. VS, vasoplegic shock.

**Table 4:** Baseline Characteristics and Outcomes According to Timing of oXiris^®^ Initiation.

Variable	oXiris^®^ ≤ 8 h (n = 16)	oXiris^®^ > 8 h (n = 14)	*p* Value
Baseline characteristics			
Age (years)	56.6 ± 22.1	67.4 ± 16.1	0.204
SOFA score	8.4 ± 1.9	8.2 ± 2.7	0.583
NE dose (μg/kg/min)	0.3 ± 0.4	0.3 ± 0.3	0.465
Lactate (mmol/L)	4.6 ± 2.4	4.1 ± 2.3	0.724
Creatinine (mg/dL)	2.0 ± 1.0	2.7 ± 2.1	0.499
CRP (mg/L)	246.1 ± 117.6	204.4 ± 82.8	0.371
Baseline cytokines			
IL-6 (pg/mL)	1053.4 ± 1419.5	440.6 ± 838.2	0.048 *
IL-1β (pg/mL)	1613.0 ± 796.2	1484.5 ± 830.1	0.724
TNF-α (pg/mL)	226.8 ± 109.4	194.8 ± 77.1	0.480
IL-10 (pg/mL)	320.1 ± 149.6	294.6 ± 134.7	0.519
Outcomes at 72 h			
VS resolution time (h) ^†^	52.6 ± 24.0	85.9 ± 48.2	0.049 *
IL-6 at 72 h (pg/mL)	373.9 ± 827.3	203.6 ± 494.5	0.114
NE dose at 72 h (μg/kg/min)	0.1 ± 0.3	0.2 ± 0.4	0.220

Data are mean ± SD. * *p* < 0.05. ^†^ Among patients with VS resolution (≤8 h: n = 12; >8 h: n = 12). IL, interleukin; NE, norepinephrine; SOFA, Sequential Organ Failure Assessment; VS, vasoplegic shock; CRP, C-reactive protein; TNF-α, tumour necrosis factor-alpha.

**Table 5:** Baseline Characteristics, Treatment Variables, and Outcomes According to 7-Day Mortality.

Variable	7-Day Survivors (n = 24)	7-Day Non-Survivors (n = 6)	*p* Value
Baseline characteristics			
Age (years)	57.9 ± 19.7	76.7 ± 13.9	0.040 *
SOFA score	8.1 ± 2.3	9.2 ± 2.2	0.317
NE dose (μg/kg/min)	0.2 ± 0.2	0.6 ± 0.6	0.014 *
Lactate (mmol/L)	3.8 ± 1.9	6.6 ± 2.4	0.016 *
Creatinine (mg/dL)	2.2 ± 1.6	2.6 ± 1.8	0.581
CRP (mg/L)	233.3 ± 111.2	199.7 ± 62.7	0.743
Baseline cytokines			
IL-6 (pg/mL)	720.1 ± 1223.8	956.7 ± 1220.3	0.561
IL-1β (pg/mL)	1632.3 ± 843.5	1236.1 ± 542.5	0.210
TNF-α (pg/mL)	217.4 ± 101.4	189.8 ± 69.7	0.392
IL-10 (pg/mL)	304.5 ± 138.2	323.3 ± 164.7	0.940
Treatment and outcomes			
Time to oXiris^®^ initiation (h)	9.4 ± 4.6	12.2 ± 12.4	0.621
Total oXiris^®^ duration (h)	108.0 ± 52.7	52.2 ± 34.7	0.017 *
IL-6 at 72 h post-oXiris^®^ (pg/mL)	104.9 ± 92.9	1052.5 ± 1352.9	0.026 *
VS resolution, n (%)	24 (100%)	0 (0%)	<0.001

Data are mean ± SD or n (%). * *p* < 0.05. All 7-day non-survivors were among patients whose vasoplegic shock did not resolve. IL, interleukin; NE, norepinephrine; SOFA, Sequential Organ Failure Assessment; VS, vasoplegic shock; CRP, C-reactive protein; TNF-α, tumour necrosis factor-alpha.

**Table 6:** IL-1β and TNF-α at 72 h After oXiris^®^ According to Hospital Mortality.

Variable	Hospital Survivors (n = 15)	Hospital Non-Survivors (n = 15)	*p* Value
IL-1β at 72 h post-oXiris^®^ (pg/mL)	856.7 ± 548.5	1086.9 ± 353.6	0.044
TNF-α at 72 h post-oXiris^®^ (pg/mL)	111.0 ± 70.0	145.4 ± 47.8	0.011

Data are mean ± SD. IL, interleukin; TNF-α, tumour necrosis factor-alpha.

## Data Availability

The data presented in this study are available on request from the corresponding author. The data are not publicly available due to privacy and ethical restrictions.

## Abbreviations

ALT	Alanine aminotransferase
AN69-ST	Polyacrylonitrile-based membrane (Baxter)
CRP	C-reactive protein
CRRT	Continuous renal replacement therapy
CVVHDF	Continuous veno-venous haemodiafiltration
ELISA	Enzyme-linked immunosorbent assay
GCS	Glasgow Coma Scale
ICU	Intensive care unit
IL-1β	Interleukin-1 beta
IL-6	Interleukin-6
IL-10	Interleukin-10
MAP	Mean arterial pressure
NE	Norepinephrine
NO	Nitric oxide
PCT	Procalcitonin
SOFA	Sequential Organ Failure Assessment
STROBE	Strengthening the Reporting of Observational Studies in Epidemiology
SVR	Systemic vascular resistance
TNF-α	Tumour necrosis factor-alpha
VS	Vasoplegic shock
WBC	White blood cell count
